# Cardiovascular Disorder Risk Assessment Among Police Personnel in Bengaluru City, India, Using World Health Organization/International Society of Hypertension Risk Prediction Chart

**DOI:** 10.7759/cureus.48378

**Published:** 2023-11-06

**Authors:** Geethu S, Jyothi Jadhav, Ranganath TS

**Affiliations:** 1 Community Medicine, Bangalore Medical College and Research Institute, Bengaluru, IND

**Keywords:** bengaluru, police personnel, who/ish risk prediction chart, cvd risk, cardiovascular event

## Abstract

Context

Cardiovascular diseases (CVDs) are important causes of premature death and disability and elevated healthcare costs. A significant percentage of this morbidity and mortality could be prevented by population-based strategies and cost-effective interventions for those at risk and with established diseases.

Aim

This study aims to estimate the 10-year risk of cardiovascular events (fatal or non-fatal) among police personnel in Bengaluru City, India.

Materials and methods

Police personnel above 40 years of age in Bengaluru City, India, were screened for CVD risk using the WHO/International Society of Hypertension (ISH) chart from November 2019 to June 2021. Data was collected by the multistage random sampling method by direct interview at the police station using a semi-structured questionnaire. CVD risk and associated factors were assessed using the WHO/ISH risk prediction chart. Data was entered into Microsoft Office Excel (Microsoft Corporation, Washington, United States) and analyzed using SPSS Statistics version 20.0 (IBM Corp. Released 2011. IBM SPSS Statistics for Windows, Version 20.0. Armonk, NY: IBM Corp.).

Results

Among 400 study participants, 9.3% (n=37) had a high risk, 2.3% (n=9) had a moderate risk, and 88.5% (n=354) had a low risk of developing fatal or non-fatal cardiovascular events in the next 10 years. Cardiovascular risk was found to be associated with certain socio-demographic and behavioral risk factors. Furthermore, a significant association (p<0.05) was found between CVD risk and the presence of comorbidities such as diabetes and hypertension.

Conclusion

The study indicates that there is a high burden of predicted cardiovascular risks among the study participants. The WHO/ISH chart can be used as a simple tool for cardiovascular risk stratification.

## Introduction

The rising burden of non-communicable diseases (NCDs) was one of the major health transitions witnessed in the 20th century. NCDs account for the deaths of 41 million people every year, which is equivalent to 71% of global deaths. It is a major cause of premature death worldwide. Around 17 million deaths due to NCDs have been reported in the age group of 30-69 years every year, of which 82% are in low- and middle-income countries (LMICs) and 37% are caused by cardiovascular diseases (CVDs) [1]. An estimated 422.7 million cases were prevalent globally in 2015 [2]. In 2016, CVDs were the leading cause of mortality worldwide and accounted for 17.9 million deaths annually, which is expected to increase to more than 23.6 million by 2030 [3].

About three-quarters of the world's deaths caused by CVDs occur in LMICs. People in LMICs often lack access to healthcare programs for early detection, especially for individuals with risk factors for CVDs, and have limited access to effective and equitable healthcare services. Therefore, the detection of diseases is often delayed throughout the course of the illness, resulting in premature deaths from CVDs and other NCDs, often occurring during the most productive years of individuals' lives.

The current epidemiological transition in India is characterized by a dual burden, with high morbidity and low mortality. This transition presents a double burden of both communicable diseases and NCDs. The prevalence of CVDs has shown a fourfold rise from 1995 to 2004 in India [4]. An estimated 2.59 million people died due to CVDs in India in 2016 [5].

The WHO defines CVDs as a group of disorders affecting the heart and blood vessels, which includes conditions such as coronary heart disease, cerebrovascular diseases, peripheral vascular diseases, rheumatic heart disease, congenital heart disease, deep vein thrombosis, and pulmonary embolism. Around 85% of CVDs are due to heart attacks and strokes [5]. Population-wide strategies such as behavioral risk factor modification can prevent most CVDs.

Law enforcement is a high-stress occupation that can significantly increase the prevalence and incidence of CVDs. Epidemiological studies highlight that police officers and public safety personnel are at an increased risk of cardiovascular morbidity and mortality. Workplace programs aimed at promoting the health and fitness of police officers are often lacking, despite being effective means for reducing cardiovascular risk.

This research attempts to assess the 10-year occurrence of cardiovascular events among police personnel in Bengaluru City using the WHO/ISH risk prediction chart. Broadly, this study addresses the burden of CVD on police personnel and explores its association with various risk factors and the predicted disease burden. The primary focus is on preventing disability, reducing early deaths from CVDs, and improving the overall quality of life among police personnel in Bengaluru City.

## Materials and methods

Study design and setting

This was a community-based cross-sectional study conducted among police personnel in Bengaluru City, India, from November 2019 to June 2021. Police personnel over 40 years of age willing to give informed consent who do not have a self-reported history of cardiovascular disorders and strokes were included in the study.

Sample size and sampling technique

Based on a previous study conducted by Premanandh et al. [6], where the prevalence of individuals at moderate to high risk of developing CVD was reported as 19.65%, and considering a relative precision (d) of 20% and a 5% type I error, the sample size was calculated to be 400.

A multi-stage random sampling technique was used to select the required number of study participants for the study. Three zones (South, West, and Central) among the five zonal divisions of law-and-order police stations were randomly selected [7]. A proportionate sampling of police stations from the respective zones was conducted. South, West, and Central zones include 20, 14, and 11 police stations, respectively. One hundred and thirty-four police officers from each zone who fulfilled the inclusion criteria were selected by simple random sampling.

Data collection

Written informed consent was obtained from all the participants. The eligible participants who fulfilled the inclusion criteria were interviewed at the police station using a semi-structured questionnaire. Data regarding socio-demographic characteristics, behavioral risk factors, and anthropometric measurements were taken from the study participants, and CVD risk and associated factors were assessed using the WHO/ISH risk prediction chart.

WHO/ISH risk prediction chart

These charts indicate a 10-year risk of fatal or non-fatal major cardiovascular events (myocardial infarction or stroke), according to age, sex, blood pressure, smoking status, total blood cholesterol, and presence or absence of diabetes mellitus for 14 WHO epidemiological sub-regions. Each chart can only be used in countries in the specific WHO epidemiological sub-region. There are two sets of charts, one with cholesterol and the other without cholesterol, for settings where cholesterol cannot be measured [8]. In our study, the SEAR-D chart without cholesterol was used.

Statistical methods

Data was entered into Microsoft Office Excel (Microsoft Corporation, Washington, United States) and analyzed using SPSS Statistics version 20.0 (IBM Corp. Released 2011; IBM SPSS Statistics for Windows, Version 20.0. Armonk, NY: IBM Corp.). Descriptive data are presented as proportions and percentages. Normally distributed quantitative data are expressed as the mean and standard deviation. Non-normal continuous data are expressed as the median and interquartile range. The chi-square test was applied to find the relationship between independent categorical variables and dependent categorical outcomes, i.e., cardiovascular risk. A p-value less than 0.05 was considered statistically significant. Data were presented in the form of tables, figures, and graphs as appropriate.

Ethical consideration

Approval and clearance were obtained from the Institutional Ethics Committee of Bangalore Medical College and Research Institute (approval number: BMCRI/PG/352/2019-20). Written informed consent was obtained from all study participants. Study objectives, procedures, benefits, risks, and anticipated use of results were explained to all participants in detail before obtaining consent.

## Results

Among the 400 study participants (Table 1), the majority of the participants, 254 (63.5%), belonged to the 40-44 age group. Males constituted 354 (88.5%) and females 46 (11.5%) of the study participants. According to religion, the majority of the study participants, 383 (95.8%), were Hindus. The majority of the study population resides in nuclear families (51.3%). Based on marital status, 96.8% were married, and about 3.2% were unmarried. About 52.8% of the study participants were educated to graduate level and above. The majority of the study participants, 296 (74%), belonged to the upper middle socioeconomic class according to the modified Kuppuswamy classification 2020. About 58 (14.5%) participants had a family history of premature coronary heart disease or stroke (Table 1).

**Table 1:** Socio-demographic profile of the study participants (n=400) *According to the modified Kuppuswamy classification 2020

Socio-demographic factors	Category	Frequency (%)
Age group (years)	40-44	254 (63.5)
	45-49	89 (22.3)
	50-54	32 (8.0)
	55-59	25 (6.2)
Sex	Males	354 (88.5)
	Females	46 (11.5)
Religion	Hindu	383 (95.7)
	Islam	16(4.0)
	Christian	1(0.3)
Type of family	Nuclear	205 (51.3)
	Joint	177 (44.3)
	Three generation	18 (4.5)
Marital status	Married	387 (96.8)
	Unmarried	13 (3.2)
Education	High school certificate	109 (27.2)
	Intermediate/diploma	80 (20.0)
	Graduate	206 (51.5)
	Profession or honors	5 (1.3)
Socioeconomic status*	Lower middle	103 (25.7)
	Upper middle	296 (74)
	Upper	1 (0.3)

On assessing the existence of comorbidities among the study participants, it was found that 4% (n=16) of the participants had hypertension, 4% (N=16) had diabetes, and 2.2% (n=9) had hypercholesterolemia.

Regarding substance abuse among the study participants, 11.5% (n=46) had the habit of smoking tobacco, 2% (n=8) had the habit of using smokeless tobacco products, and 17.2% (n=69) had the habit of consuming alcohol. On assessing the consumption of fruits and vegetables, the majority of the study participants, 368 (92%), consumed less than five combined servings of fruits and vegetables per day. The majority of the study participants, 93% (n=373), performed moderate physical activity, 3% (n=12) performed high physical activity, and 4% (n=15) performed only low physical activity.

The physical measurements of the participants were also taken to assess their association with cardiovascular risk (Table 2).

**Table 2:** Physical measurements of the study population IQR: interquartile range, BMI: body mass index

Variables	Median	IQR
Height (cm)	171	169-174
Weight (kg)	76	69-84
BMI (kg/m^2^)	26.12	23.46-28.41
Waist (cm)	84	82-85
Hip (cm)	99	97-102
Waist-hip ratio	0.84	0.83-0.87
Systolic blood pressure (mm of Hg)	131	120.25-145.2
Diastolic blood pressure (mm of Hg)	79.00	73.75-84.00
Heart rate (beats per minute)	78	74-88
Random blood sugar (mg/dl)	105.00	87-130

Overall cardiovascular risk of the study participants

Among the 400 police personnel, it was found that 88.4% (n=354) had low risk, 2.3% (n=9) had moderate risk, and 9.3% (37) had a high risk of developing a cardiovascular event in 10 years.

Association between participant characteristics and CVD risk

The association between cardiovascular risk and various socio-demographic and behavioral factors and physical measurements was assessed using a chi-square test (Tables 3-4). Among the socio-demographic factors, age, marital status, level of education, and socioeconomic status had a significant association with cardiovascular risks.

**Table 3:** Risk factors in the study participants * A p-value less than 0.05 is considered statistically significant

Variables	Status	Cardiovascular risk			χ2	p-value
		Low n (%)	Moderate (%)	Severe (%)		
Hypertension	Absent	345 (89.8)	9 (2.3)	30 (7.8)	23.78	<0.01*
	Present	9 (56.2)	0 (0.0)	7 (43.8)		
Diabetes mellitus	Absent	346 (90.1)	4 (1.0)	34 (8.9)	43.23	<0.01*
	Present	8 (50.0)	5 (31.2)	3 (18.8)		
Hypercholesterolemia	Absent	347 (88.7)	8 (2.0)	36 (9.2)	3.367	0.18
	Present	7 (77.8)	1 (11.1)	1 (11.1)		
Smoking tobacco	Absent	327 (92.4)	3 (0.8)	24 (6.8)	52.43	<0.01*
	Present	27 (58.7)	6 (13.0)	13 (28.3)		
Smokeless tobacco	Absent	348 (88.8)	9 (2.3)	35 (8.9)	2.54	0.28
	Present	6 (75.0)	0 (0.0)	2 (25.0)		
Alcohol	Absent	295 (89.1)	5 (1.5)	31 (9.4)	4.77	0.09
	Present	59 (85.5)	4 (5.8)	6 (8.7)		
Fruit and vegetable servings per day	<5	329 (89.4)	7 (1.9)	32 (8.7)	4.42	0.10
	>5	25 (78.2)	2 (6.2)	5 (15.6)		
Physical activity in MET minutes	Low	13 (86.7)	1 (6.7)	1 (6.7)	3.06	0.548
	Moderate	329 (88.2)	8 (2.1)	36 (9.7)		
	High	12 (100)	0 (0)	0 (0)		

A significant association was found between the presence of diabetes mellitus and/or hypertension and cardiovascular risk. The current study did not show any significant association between physical activity or consumption of fruits and vegetables and cardiovascular risk. The use of smokeless tobacco products and the consumption of alcohol did not have a statistically significant association with cardiovascular risk patterns (Table 3).

Among the physical measurements, systolic blood pressure was found to have a significant association with CVD risk (Table 4).

**Table 4:** Association between physical measurements and CVD risk score categories * A p-value less than 0.05 is considered statistically significant, BMI: body mass index

Variable	Variable category	Cardiovascular risk			χ2	p-value
		Low n (%)	Moderate n (%)	Severe n (%)		
BMI	Underweight	5 (83.3)	0 (0.0)	1 (16.7)	1.48	0.96
	Normal	128 (90.1)	2 (1.4)	12 (8.5)		
	Overweight	194 (87.8)	6 (2.7)	21 (9.5)		
	Obese	27 (87.1)	1 (3.2)	3 (9.7)		
Systolic blood pressure	<140 mm of Hg	263 (99.6)	1 (0.4)	0 (0.0)	94.75	<0.01*
	≥140 mm of Hg	91 (66.9)	8 (5.9)	37 (27.2)		
Waist-hip ratio	Normal	333 (87.9)	9 (2.4)	37 (9.8)	2.88	0.237
	Raised	21 (100.0)	0 (0.0)	0 (0.0)		

## Discussion

Among the 400 police personnel, on estimating the CVD risk using the WHO/ISH risk prediction chart without cholesterol, it was found that 9.3% had a high risk, 2.3% had a moderate risk, and 88.5% had a low risk of developing cardiovascular events in the next 10 years.

Several studies suggest that police officers and other public safety personnel have a higher risk of cardiovascular morbidity and mortality. Law enforcement activities were often found to be stressful and physically demanding and associated with a higher risk of sudden cardiac death compared to routine non-emergency occupations [9]. There is limited epidemiological data on the morbidity profile and risk assessment of CVDs among police personnel in India. A study by Meena et al. found a high prevalence of 36.2% morbidity associated with metabolic and cardiovascular risks in the National Capital Region of India [10]. In addition, a study conducted to evaluate the cardiovascular risk factors of police personnel in West Bengal, India, found that police personnel (38.2%) suffered from more cardiovascular risk factors compared to non-police personnel (21.2%) [11]. Studies using the WHO/ISH risk prediction tool were not conducted among police personnel to assess cardiovascular risk patterns previously in India.

The measurement and monitoring of population-level total cardiovascular risk using the WHO/ISH risk prediction chart is an effective method of assessing the total preventive needs and effective interventions required by multiple CVD risk factors in different magnitudes and directions [12]. Several studies have been conducted among the general population of India to assess CVD risk using the WHO/ISH chart. A study by Norman et al. conducted in 47 villages in Karnataka over three years found a 28%, 12.8%, and 58.1% high, moderate, and low risk of developing CVD in the study population, respectively [13]. A study in Pondicherry by Ghorpade et al. found a 10.2%, 6.8%, and 83% high, moderate, and low risk of developing CVD in the study population, respectively [14]. A similar study by Gill et al. in Amritsar found a high risk of 28.8%, a moderate risk of 15.5%, and a low risk of 55.8% among the study participants [15]. The WHO/ISH chart risk prediction charts have been used worldwide for the estimation of cardiovascular risk, and varied proportions of risk have been found across populations.

A study by Nordet et al. in Havana, Cuba, found a high risk of 4.6%, a moderate risk of 12.7%, and a low risk of 82.7% in the study population [16]. A similar study by Dhungana et al. in Kathmandu, Nepal, found 5.4%, 9.1%, and 85.5% of high, moderate, and low risks of developing CVD in the study population [17]. A study by Babatunde et al. in Nigeria showed that the proportion of respondents with a low, moderate, and high risk of developing CVDs within 10 years was 76.9%, 8.5%, and 14.6%, respectively [18].

Association between socio-demographic characteristics and predicted cardiovascular risk

In the current study, a significant association (p<0.05) was found between age and cardiovascular risk patterns. Age is a well-established predictor of cardiovascular risk; it is similar to the results by Norman et al., Gill et al., and Dhungana et al. The reason for this consistent association is that as age increases, the prevalence of significant risk factors, such as diabetes and hypertension, also increases. Consequently, the predisposition to develop CVDs also becomes higher [13,15,17].

The present study did not demonstrate any significant association between gender and cardiovascular risk, although evidence from several epidemiological studies has shown a favorable mortality trend in females compared to males [19].

A statistically significant association was found between the marital status of the individuals and their cardiovascular risk. Unmarried study participants were more at risk of cardiovascular events compared to married individuals. A systematic review and meta-analysis by Wong et al. found that, compared to married participants, being unmarried (never married, divorced, or widowed) was associated with increased odds of CVD [20]. Similar observations were found in the study by Manfredini et al. [21].

A statistically significant association was also found between the level of education and CVD risk in the present study. The findings from a prospective cohort study by Christensen et al., which aimed to assess the relationship between the level of education and the risk of heart failure, revealed that the age-adjusted hazard ratio (HR) was higher among those educated up to the intermediary level, at 0.69 (0.62-0.78), compared to individuals with a higher level of education, at 0.52 (0.43-0.63) [22]. The inverse relationship between the level of education and cardiovascular risk was also demonstrated by Degano et al., where a higher CVD incidence hazard ratio was found in those with primary or lower education compared to those who have completed degree-level education, 0.51 (95% CI=0.30, 0.85) [23]. The association may be due to mediating modifiable risk factors such as hypertension, diabetes, dyslipidemia, behavioral factors, BMI, and physical activity in the population.

A statistically significant association was also found between socioeconomic status and cardiovascular risk in the present study. An inverse relationship between socioeconomic status and CVD risk patterns was also demonstrated in a study by Kozakiewicz K et al. [24]. A study by Subramanian et al. found a higher CVD-related mortality rate among lower socioeconomic status groups, whereas the proportion of deaths from CVD-related causes was found to be the greatest among higher socioeconomic status groups [25].

A statistically significant association (p=0.04) was found in the current study between a family history of premature coronary heart disease or stroke in first-degree relatives and the predicted cardiovascular risk. The CVD risk from family history depends on the number of first-degree relatives affected and also the age at which CVD developed. Furthermore, a study among the offspring of the Framingham study showed a 75% increased risk with paternal and a 60% increased risk with a maternal history of premature CVD [26].

A statistically significant association (p<0.05) was found between the existence of diabetes and hypertension and the predicted risk of cardiovascular disease. Multiple studies have shown that people with diabetes mellitus have a two-to-four-fold increase in risk of incident coronary heart disease and ischemic stroke and a 1.5-to-3.6-fold increase in mortality [27]. Most population-based studies confirm that hypertension increases an individual’s risk of various cardiovascular consequences approximately two to three times [28]. Hypercholesterolemia is a well-documented risk factor for cardiovascular disease. The diagnosis and management of hyperlipidemia have been primary methods of preventing CVD by primary healthcare physicians. The current study did not show any statistically significant association between pre-existing hypercholesterolemia and the estimated CVD risk.

Many previous studies have demonstrated higher all-cause mortality among overweight individuals compared to those with a normal BMI. The current study did not show any statistically significant association between BMI and predicted CVD risk. In a US prospective cohort study, it was found that compared to individuals with normal weight, middle-aged men and women had competing hazard ratios for incident CVD of 1.21 (95% CI, 1.14-1.28) and 1.32 (95% CI, 1.24-1.40) for overweight, 1.67 (95% CI, 1.55-1.79) and 1.85 (95% CI, 1.72-1.99) for obesity, and 3.14 (95% CI, 2.48-3.97) and 2.53 (95% CI, 2.20-2.91) for morbid obesity, respectively. Higher BMI had the strongest association with incident heart failure among CVD subtypes [29].

Random plasma glucose values of the study participants were also measured, which helped to assess the overall CVD risk using the WHO/ISH chart. Previous studies have shown that increasing levels of plasma glucose are associated with increasing lipid levels in serum, as well as with a higher BMI and systolic blood pressure. This clustering of risk factors may lead to the future development of coronary events in the population [30].

Thus, the present study highlighted the level of risk of developing a fatal or non-fatal cardiovascular event among police personnel in Bengaluru City. Overall, 9.3% of the study participants were at high risk of developing CVD. The study also showed a statistically significant association between CVD risk and factors such as age, marital status, level of education, family history of premature coronary heart disease, smoking tobacco, comorbidities such as diabetes and hypertension, and systolic and diastolic blood pressure measured. Therefore, preventive strategies and policies can be directed at reducing these modifiable risk factors identified, which can aid in the overall reduction of cardiovascular risk.

Certain limitations were noted during the course of the study. Due to resource limitations in the study setting, the WHO/ISH charts with cholesterol could not be utilized for the study. In addition, there is a possibility of reporting and recall bias while assessing self-reported risk factors. Since the study tool is a screening tool designed to predict cardiovascular risk, further detailed diagnostic workups are necessary.

## Conclusions

WHO/ISH risk prediction charts and the accompanying guidelines are effective tools for cardiovascular risk prediction and management, even in settings that do not have sophisticated technology. The findings from the present study predicted the possibility of the occurrence of cardiovascular events among police personnel and identified possible risk factors associated with them. A comprehensive risk approach along with periodic screening can be an effective method of preventing the emergence of cardiovascular events in the long term.
